# Comparative Volumetric Analyses Following Bevacizumab Therapy for a Patient With Concomitant Glioblastoma, Meningioma, and Dural Arteriovenous Fistula: A Case Report and Review of Literature

**DOI:** 10.7759/cureus.69794

**Published:** 2024-09-20

**Authors:** Akihiko Teshigawara, Tomoto Kyoichi, Yuzuru Hasegawa, Yuichi Murayama, Toshihide Tanaka

**Affiliations:** 1 Neurosurgery, The Jikei University School of Medicine, Kashiwa Hospital, Kashiwa, JPN; 2 Neurosurgery, The Jikei University School of Medicine, Tokyo, JPN

**Keywords:** vascular endothelial growth factor, dural arteriovenous fistula, meningioma, glioblastoma, neoadjuvant bevacizumab

## Abstract

Given that glioblastoma (GBM), meningioma (Mg), and dural arteriovenous fistula (dAVF) represent angiogenic diseases mainly caused by vascular endothelial growth factor (VEGF), bevacizumab (Bev) is expected to be effective against these diseases. We report a patient with concomitant GBM, Mg, and dAVF who was treated with neoadjuvant Bev, resulting in a reduction in the volume of GBM along with an improvement of clinical symptoms.

An 85-year-old male presented with aphasia, gait disturbance, and dementia. Magnetic resonance imaging (MRI) showed a ring-enhanced intra-axial tumor with perifocal edema in the left temporal lobe, a dura-attached extra-axial tumor at the left sphenoid ridge, and dAVF at the left transverse-sigmoid sinus. Due to the age of the patient and low Karnofsky Performance Status (KPS) score, pharmacotherapy with a single dose of Bev was chosen over surgical resection. Three days after the Bev administration, aphasia and gait disturbance had dramatically improved. Volume reduction rates at one and five months after three administrations of Bev were 0.34% and 95.9% for GBM and 13.7% and 6.8% for meningioma, respectively. No significant change in dAVF was seen on digital subtraction angiography (DSA) during Bev therapy.

VEGF concentration in GBM is known to be the highest among all types of brain tumors, including meningioma. VEGF might not play a pivotal role in the pathogenesis of dAVF. Based on this evidence from the present rare case with concomitant GBM, meningioma, and dAVF, responsiveness to Bev might depend on the level of VEGF expression.

## Introduction

Although several molecular mechanisms contribute to tumor angiogenesis, the vascular endothelial growth factor (VEGF) pathway seems particularly important and has thus served as a prominent therapeutic target in cancer treatment [1,2].

Anti-VEGF therapy with the monoclonal antibody bevacizumab (Bev) and the tyrosine kinase inhibitors sorafenib and sunitinib has demonstrated clinical benefit in randomized clinical trials involving patients with various solid tumor types and has already been approved by the Food and Drug Administration in the United States.

In the treatment of various cancers, anti-VEGF therapy has been included in the standard therapeutic protocol for hypervascular cancers such as renal cell carcinoma (RCC), colorectal cancer with liver metastases, ovarian cancer with ascites, and non-small cell lung cancer. RCC is one of the most hypervascular tumors, and overexpression of VEGF is well known as the most important predisposing factor for disease progression [3].

Bev has been approved for both newly diagnosed and recurrent glioblastoma (GBM) as a standard therapeutic protocol in Japan. In the course of daily Bev use, we have explored the possibility of using Bev in neoadjuvant settings either as a safer alternative to surgical resection or as a consequence of the clinical course. In those cases, we achieved significant reductions in tumor vascularity and brain swelling without particular adverse events, and we conducted a prospective multi-institutional phase II clinical study for newly diagnosed GBM to confirm the safety and clinical benefit of Bev [4].

VEGF plays a pivotal role in highly vascular tumors other than GBM, including meningioma (Mg) [5]. The safety and therapeutic efficacy of Bev therapy for atypical and progressive meningioma were evaluated. Median progression-free survival (PFS) and PFS rate at six months were 6.5-18 months and 43%-86%, respectively [6,7].

Given that VEGF expression was detected in 64.5% of meningiomas, relationships between the level of VEGF expression and characteristics of meningioma including expanding perifocal edema, recurrence rate, growth rate for aggressiveness, and histological grade of malignancy remain controversial [8]. While meningioma is known as a hypervascular tumor, the concentration of VEGF in meningioma tissues was significantly lower than that in GBM [9].

In other vascular diseases including arteriovenous malformation and dural arteriovenous fistula (dAVF), VEGF might play key roles in pathogenesis and as a therapeutic target. Angiogenic factors may contribute to the development of dAVF [10,11]. Tissue hypoxia or intraluminal shear stress caused by venous hypertension might induce the expression of VEGF as evidenced by in situ investigation using surgical samples obtained from patients with dAVF [10,12,13]. In addition, other reports have noted that the degree of improving VEGF expression corresponded to the degree of recovery for the patient with dAVF, whereas VEGF expression in patients with dAVF would seem to increase as deterioration or refractoriness [14].

Theoretically, GBM, meningioma, and dAVF represent angiogenic diseases mainly caused by high expression of VEGF, so Bev might be expected to be effective. Given that Bev is approved only for patients with GBM, we cannot guarantee its effectiveness against meningioma and dAVF due to a lack of experience.

To address the issue of whether Bev is effective for meningioma and dAVF as for GBM, we relate our experience with a patient with concomitant GBM, meningioma, and dAVF who was treated using neoadjuvant Bev as a first-line therapeutic option, resulting in volume reduction of GBM with improvement of clinical symptoms.

## Case presentation

Clinical course and neuroradiological findings before and after neoadjuvant Bev

**Figure 1:**
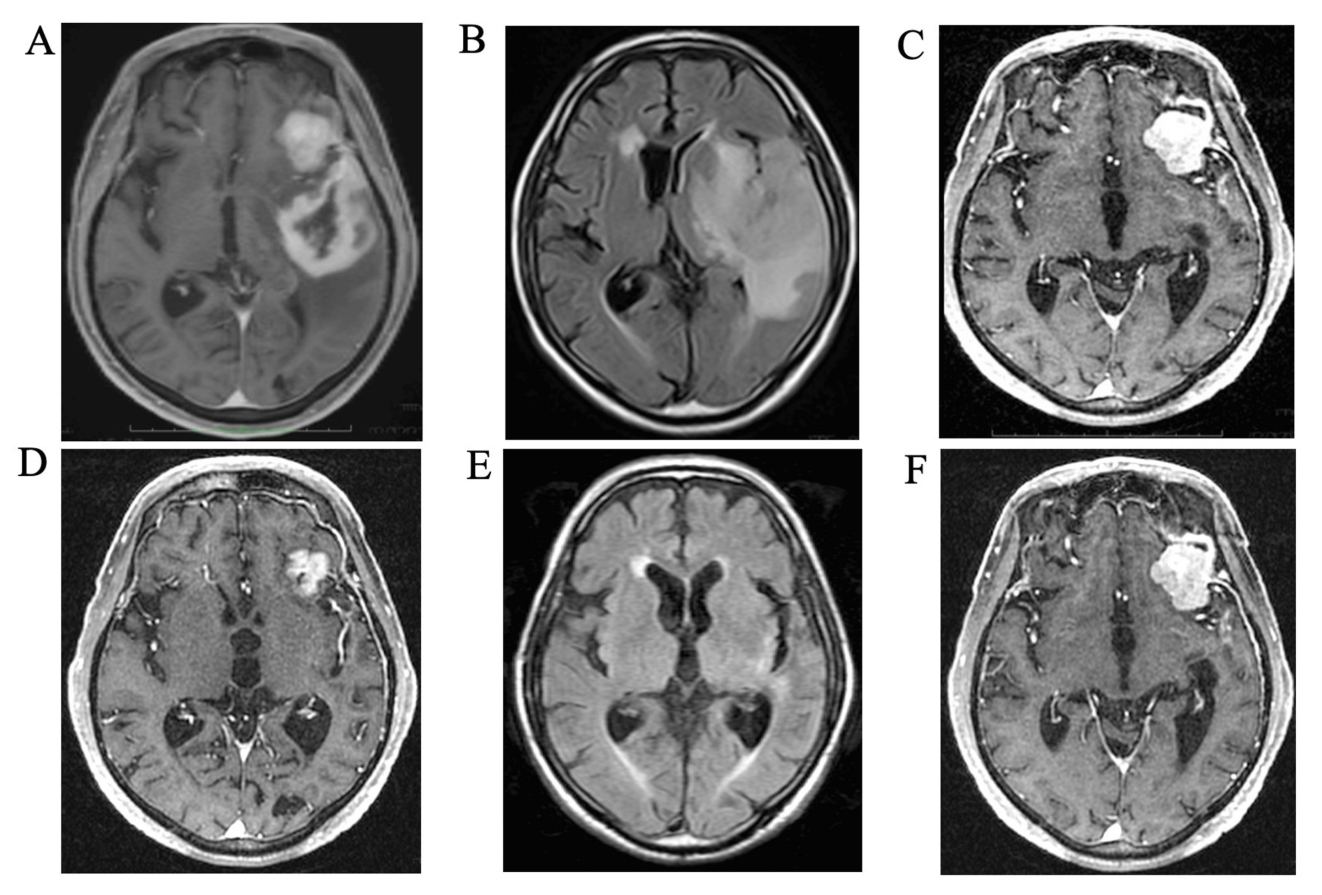
MRI with Gd enhancement (T1-Gd) (A) Initial T1-weighted MRI with Gd enhancement (T1-Gd) shows a large intra-axial tumor with ring enhancement. (B) FLAIR MRI shows the intra-axial tumor causing brain edema around the tumor. (C) T1-Gd shows a large extra-axial tumor attached to the dura with homogeneous enhancement. (D) T1-Gd five months after Bev administration shows disappearance of enhanced lesion of the intra-axial tumor and a slight reduction in the size of the extra-axial tumor. (E) FLAIR MRI shows disappearance of expanding perifocal edema surrounding the tumor. (F) Compared with (C), T1-Gd five months after Bev administration shows no remarkable differences in the volume of the extra-axial tumor. MRI: magnetic resonance imaging, Gd: gadolinium, FLAIR: fluid-attenuated inversion recovery, Bev: bevacizumab

An 85-year-old male had suffered from aphasia and dementia, with a gradual loss of his ability to walk. Finally, he had to be hospitalized. Surprisingly, T1-weighted magnetic resonance imaging (MRI) with gadolinium enhancement (T1-Gd) and fluid-attenuated inversion recovery (FLAIR) imaging showed three lesions: intra-axial tumor in the left temporal lobe (Figure 1A, 1B) and extra-axial tumor attached to the dura at the left sphenoid ridge (Figure 1C). T1-Gd helped with differential diagnosis, demonstrating that the intra- and extra-axial tumors were likely GBM and Mg, respectively. GBM in the left temporal lobe revealed robust perifocal edema causing severe neurological symptoms such as aphasia and hemiparesis in the right extremities.

First, he was treated with a single administration of Bev as neoadjuvant therapy, since surgical resection for both GBM and Mg was considered too invasive based on patient age along with low Karnofsky Performance Status (KPS) score and the tumor location.

Three days after 10 mg/kg of initial Bev administration, aphasia and gait disturbance had improved dramatically. One and a half months later, he was discharged home. T1-Gd and FLAIR imaging after Bev therapy demonstrated a dramatic decrease and disappearance in the volume of the intra-axial tumor (Figure 1D, 1E), whereas the volume of the extra-axial tumor had not changed (Figure 1F).

Neuroradiological assessment for dAVF

**Figure 2:**
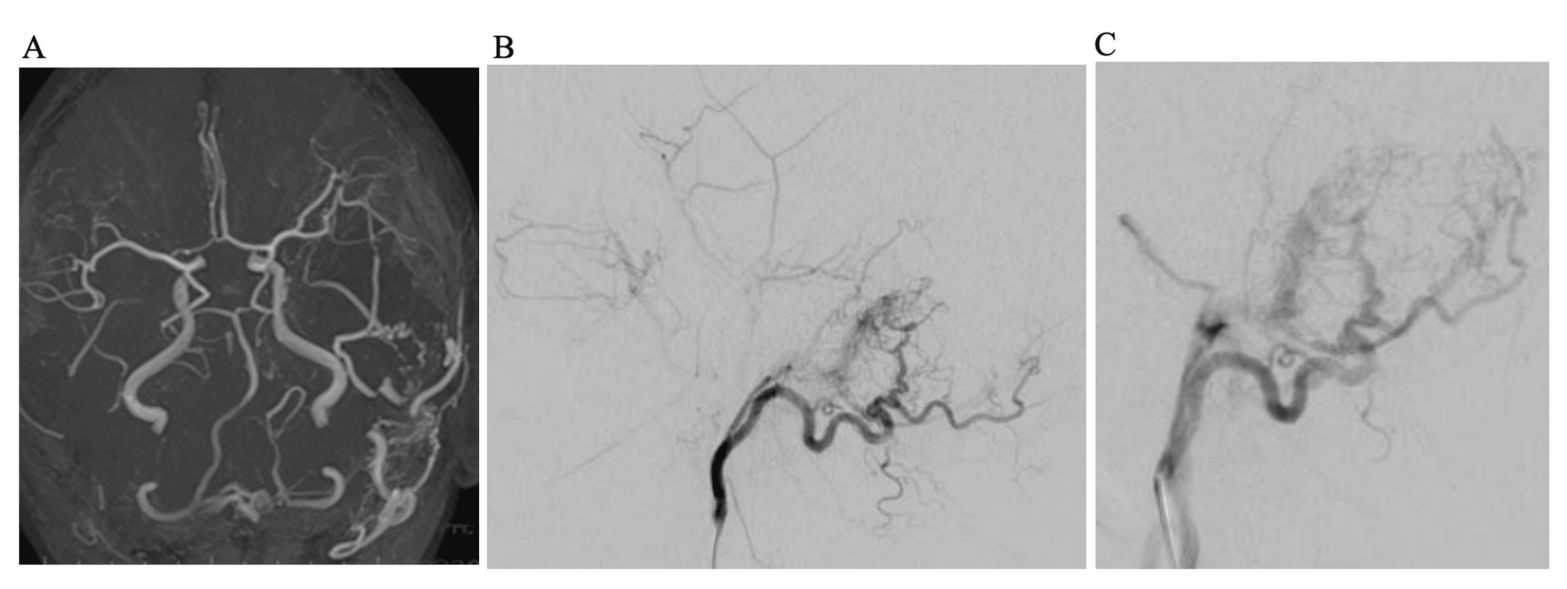
MRA before and after Bev therapy (A) MRA reveals dAVF comprising abnormal vasculature originating from the external arteries at the left transverse sinus. (B) Lt-ECG before Bev therapy shows dAVF at the left sigmoid sinus. Feeders from the OA, posterior auricular artery, middle meningeal artery, and STA head to the shunt point. Shunt blood flow runs through the ipsilateral sigmoid sinus to the IJV in an antegrade manner and through the oblique occipital sinus to the contralateral transverse sinus in a retrograde manner. (C) Lt-ECG one month after Bev administration reveals no change in dAVF hemodynamics compared with the initial Lt-ECG (B). MRA: magnetic resonance angiography, Bev: bevacizumab, dAVF: dural arteriovenous fistula, Lt-ECG: left external carotid angiography, OA: occipital artery, STA: superficial temporal artery, IJV: internal jugular vein

In addition, an arteriovenous shunt was present at the dura along the left transverse sigmoid sinus (Figure 2A). Digital subtraction angiography (DSA) was performed twice, before and after neoadjuvant Bev (Figure 2B, 2C). Magnetic resonance angiography (MRA) for dAVF assessment had also been performed every month.

DSA on admission showed dAVF at the left transverse-sigmoid sinus. The feeders were the left occipital artery (OA), posterior auricular artery, superficial temporal artery, and middle meningeal artery. Shunt blood flow ran through the ipsilateral sigmoid sinus to the internal jugular vein (IJV) in an antegrade manner and through the oblique occipital sinus to the contralateral transverse sinus in a retrograde manner without cerebral venous reflux (Cognard type IIa+b) (Figure 2B). These findings remained unchanged one month after Bev administration (Figure 2C). MRA findings also remained largely unchanged every month.

Follow-up after neoadjuvant Bev

Tumor resection was initially planned for after Bev administration, but the patient and his family decided against surgery because of his remarkable recovery. Instead, he opted to continue receiving Bev every month. However, this was discontinued because of an allergic reaction to Bev with the third administration. He was followed up by course observation with regular neuroimaging on an outpatient basis.

Volumetric assessments before and after neoadjuvant Bev

To compare reactivity to Bev among these three pathologies, we undertook a volumetric quantitative assessment of GBM and Mg using MRI with gadolinium enhancement before and after Bev administration. Using a Vincent platform (Fujitsu, Japan), we calculated the volume of the enhanced region of tumors at the time of hospitalization and every month for five months after Bev administration.

Alteration of intra-axial tumor volume during Bev therapy

**Figure 3:**
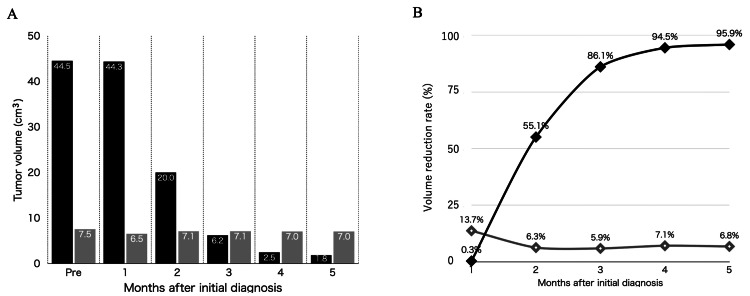
Alterations of tumor volume reduction rate during Bev therapy Alterations of tumor volume (A) and volume reduction rate (B) in GBM and Mg during Bev therapy. GBM tumor volume continuously decreased after Bev administration, whereas that of Mg did not significantly decrease during follow-up up to five months after initial diagnosis. Black square and black rhombus: GBM, grey square and open rhombus: Mg Bev: bevacizumab, GBM: glioblastoma, Mg: meningioma

The initial tumor volume was 44.5 cm^3^. Tumor volumes at one, two, three, four, and five months after Bev administration were 44.3 cm^3^, 20.0 cm^3^, 6.2 cm^3^, 2.5 cm^3^, and 1.8 cm^3^, respectively (Figure 3A). After five months, the volume reduction rate was 95.9% (Figure 3B).

Alteration of extra-axial tumor volume during Bev therapy

The initial tumor volume was 7.5 cm^3^. Tumor volumes at one, two, three, four, and five months after Bev administration were 6.5 cm^3^, 7.1 cm^3^, 7.1 cm^3^, 7.0 cm^3^, and 7.0 cm^3^, respectively (Figure 3A).

The pattern of volume reduction for Mg thus differed from that for GBM. In the first month, the volume decreased by 13.7%, exceeding the decrease seen in GBM. Thereafter, the volume reduction rate plateaued at about 6%-7% (Figure 3B).

## Discussion

Six mechanisms of tumor vessel formation have been postulated: vasculogenesis, sprouting angiogenesis, vessel co-option, intussusception, vascular mimicry, and transdifferentiation of tumor cells into endothelial cells. These modes of new vessel formation may be regulated by VEGF, along with myriad other molecules and signal transduction pathways.

GBM and VEGF

VEGF is believed to play a bifunctional role in malignant brain tumor biology leading to both angiogenesis and vasogenic edema, regulating the formation of new blood vessels and their permeability, acting through VEGF receptor (VEGFR)-1 and VEGFR-2.

Takano et al. determined the concentration of VEGF in sera and tumor extracts from 19 patients with brain tumor, including GBM, anaplastic astrocytoma, low-grade astrocytoma, meningioma, malignant lymphoma, and metastatic brain tumor, as well as normal brain [9]. They found that VEGF concentrations were significantly higher in GBM tissues than in other tumor types or normal brain. Increased levels of VEGF expression would result in highly angiogenic tumors leading to abnormal vasculature.

The effectiveness of Bev is well known for recurrent GBM. We think that for older patients with GBM in particular, Bev could be a key drug for achieving radiographic improvement and improving or preventing deterioration of symptoms with fewer adverse effects. Neoadjuvant chemotherapy, as upfront administration of Bev before tumor resection, could benefit patients by improving symptoms, decreasing tumor volume, and reducing the expansion of perifocal edema [9]. In the present case, neoadjuvant Bev therapy had sufficient effect against GBM that operation was considered unnecessary.

Meningioma and VEGF

Meningioma is known as a hypervascular tumor. To reduce tumor vascularity, preoperative embolization of meningioma has been performed, although the effectiveness of preoperative embolization remains controversial. Tumor blood volumes have been compared between pre- and post-embolization for patients with Mg. The post-embolization normalized tumor blood volume was significantly lower than that of pre-embolization according to dynamic susceptibility contrast-enhanced perfusion-weighted imaging. Gruber et al. also showed that the postoperative vascularity of supratentorial hemispheric meningioma decreased to about 60%, compared with preoperative embolization for tumor vessels using microsphere [15].

VEGF plays an important role in angiogenesis in meningioma, particularly with the degree of vasculature observed angiographically. Winter et al. detected that VEGF expression was positive in 64.5% of meningiomas in their cohort series [8]. In general, the expression levels of VEGF tend to be higher in atypical and anaplastic meningiomas or recurrences associated with expanding perifocal edema. A multi-institutional phase II trial of Bev for recurrent and refractory meningioma found that PFS at six months, median PFS, and median OS were 87%, 22 months, and 35 months for grade 1 meningioma; 77%, 23 months, and 41 months for grade 2 meningioma; and 46%, eight months, and 12 months for grade 3 meningioma [6]. They concluded that Bev appears to hold promise as a next-line therapy after surgery and radiation for recurrent and refractory meningioma.

On the other hand, other studies have found no correlation between the degree of VEGF expression and tumor size, its location, histopathological subtype, surrounding edema, recurrence rate, growth rate, and tumor grade, with no correlation between the amount of VEGF and tumor vascularization or histological grade of malignancy [8]. In addition, the concentration of VEGF was significantly lower in meningioma tissues than in GBM tissues [9].

Given the effect of Bev against meningioma, especially recurrent and high-grade cases, a systematic review concluded that patients with recurrent meningioma do not seem to benefit significantly from Bev monotherapy. Further, tumor response does not correlate with any known patient or tumor characteristics or image analyses, including dynamic contrast-enhanced MRI kinetic parameters.

Regardless of tumor hypervascularity, angiogenesis in meningioma might not be VEGF-dependent and is likely heterogeneous among cases, compared with malignant gliomas, probably due to the degree to which the tumor microenvironment includes areas of hypoxic conditions and recruitment of immunoregulatory cells.

dAVF and VEGF

Some angiogenic factors including VEGF may contribute to the development of dAVF [10,12,16]. Tissue hypoxia or intraluminal shear stress resulting from venous hypertension upregulates VEGF, and the expression of VEGF has been identified in surgical specimens from dAVF patients [13]. A previous report suggested that cerebral ischemia occurs due to venous congestion in dAVF and raised VEGF levels in the serum [17].

In contrast, it remains unknown whether high expression of VEGF is pivotal to the formation of dAVF. VEGF does not appear to be a major factor for the development of dAVF but rather a secondary effect. In addition, other factors associated with angiogenesis and invasion, such as platelet-derived growth factor (PDGF), transforming growth factor, Ephrin, and matrix metalloproteases, are also important in the process of angiogenesis and vascular remodeling [18]. As a result, the upregulation of VEGF expression levels alone may not be able to significantly increase the rate of dAVF induction [14].

The effect of Bev on concomitant GBM and non-neoplastic disease

To the best of our knowledge, no previous reports have clarified the effects of Bev on patients with concomitant GBM and other neoplasms and non-neoplastic diseases, including meningioma and dAVF. One case report described recurrent GBM after standard therapy accompanied by chronic subdural hematoma (CSDH), demonstrating that CSDH as well as edema surrounding the tumor completely disappeared after Bev therapy [19]. The rationale for the effect of Bev on CSDH was based on evidence that the outer membrane of the CSDH contained high levels of VEGF and the hematoma in the subdural space showed a high concentration of VEGF, leading to the accumulation of subdural fluid via vascular permeability in the angiogenic microvasculature [19].

For appropriate treatment of concomitant GBM, meningioma, and dAVF, surgical resection for GBM and meningioma and interventional endovascular surgery for dAVF should be considered in general. Salvage therapy with Bev after standard therapy might be necessary, particularly in the event of recurrence or refractory disease. For elderly patients with low KPS, neoadjuvant Bev therapy for angiogenic diseases such as hypervascular tumors or vascular malformation might be an alternative therapeutic strategy, particularly in rare cases of different diseases coinciding in the same patient.

Limitations

A key limitation of the present study was that the patient did not undergo surgical resection of the tumors and dAVF due to poor preoperative KPS score. Regardless of the dramatic improvement after Bev therapy, the patient declined further surgical interventional therapy. We, thus, lost an opportunity to confirm the histological diagnoses of the three concomitant lesions and the expression of VEGF in tumor tissues and vascular lesions. Findings including the level of VEGF expression in each lesion could be speculated from the response to Bev therapy.

## Conclusions

The present study verified that neoadjuvant therapy with anti-VEGF antibody can offer a useful alternative therapeutic strategy for meningioma and GBM but can be controversial for dAVF. Ideally, surgical resection, biopsy, or serum collection could be considered to quantify concentrations of VEGF and judge whether neoadjuvant Bev therapy is likely to offer clinical benefit.

Based on this evidence from a patient with concomitant GBM, meningioma, and dAVF, we gained insights into the pathogenesis of three different "highly vascular diseases" related to the expression of VEGF/VEGFRs.
